# MCR-5-Producing Colistin-Resistant Cupriavidus gilardii Strain from Well Water in Batna, Algeria

**DOI:** 10.1128/mSphere.00575-21

**Published:** 2021-09-01

**Authors:** Zineb Cherak, Lotfi Loucif, Mariem Ben Khedher, Abdelhamid Moussi, Amel Benbouza, Sophie Alexandra Baron, Jean-Marc Rolain

**Affiliations:** a Laboratoire de Génétique, Biotechnologie et Valorisation des Bio-ressources (GBVB), Faculté des Sciences Exactes et des Sciences de la Nature et de la Vie, Université Mohamed Khider, Biskra, Algeria; b Laboratoire de Biotechnologie des Molécules Bioactives et de la Physiopathologie Cellulaire (LBMBPC), Faculté des Sciences de la Nature et de la Vie, Université de Batna 2, Batna, Algeria; c Aix Marseille Univ., IRD, APHM, MEPHI, IHU-Méditerranée Infection, Marseille, France; d IHU Méditerranée Infection, Marseille, France; e Assistance Publique des Hôpitaux de Marseille, Marseille, France; f Faculté de Médecine, Université de Batna 2, Batna, Algeria; Antimicrobial Development Specialists, LLC

**Keywords:** *Cupriavidus gilardii*, *mcr-5*, colistin resistance, groundwater, Algeria

## Abstract

This paper presents the first description of the *mcr-5.1* gene in a colistin-resistant Cupriavidus gilardii isolate from well water that supplies a maternity hospital in Algeria. The whole-genome sequence of this strain showed the presence of putative β-lactamase, *aac(3)-IVa*, and multidrug efflux pump-encoding genes, which could explain the observed multidrug resistance phenotype. Our findings are of great interest, as we highlight a potential contamination route for the spread of *mcr* genes.

**IMPORTANCE** Colistin resistance mediated by *mcr* genes in Gram-negative bacteria has gained significant attention worldwide. This is due to the ability of these genes to be horizontally transferred between different bacterial genera and species. Aquatic environments have been suggested to play an important role in the emergence and spread of this resistance mechanism. Here, we describe the first report of an *mcr-5*-positive Cupriavidus gilardii aquatic isolate through its isolation from well water in Algeria. The significance of our study is in shedding the light on an important environmental reservoir of *mcr* genes.

## OBSERVATION

Since the first detection of the plasmid-mediated colistin resistance mechanism in December 2015, 10 *mcr* genes and several variants have been identified worldwide from different sources (1, 2). Being transferable, this mechanism has received more attention than any of the colistin resistance mechanisms previously described. Indeed, the origin of this mechanism has long preoccupied researchers, and different studies have suggested an environmental origin, particularly an aquatic one (3–5), which could participate significantly in its dissemination to pathogenic bacteria. Likewise, aquatic environments can act as an important vehicle for the spread of such resistance mechanisms to humans either in the community or, more worryingly, in hospital settings.

In this paper, we present the first report of the *mcr-5* gene in an unusual bacterial isolate, Cupriavidus gilardii, recovered from well water that supplies a maternity hospital in the Batna province, Algeria.

During September and October 2019, 38 water samples were obtained from a maternity hospital in Batna city, Algeria. The hospital is located in an urban region where no agricultural activity is near the study site. One liter of water was collected in sterile glass bottles from the well that supplies the hospital with tap water, from water tanks, and from taps with the hospital’s various wards. Each water sample was filtered through a cellulose membrane (0.45 μm pore size), and the filter was placed on a MacConkey agar plate (HiMedia, India). Plates were incubated overnight aerobically at 37°C. Cultures were purified and identified using matrix-assisted laser desorption ionization–time of flight mass spectrometry (6). Thereafter, isolates were screened by real-time PCR for the occurrence of *mcr-1*, *mcr-2*, *mcr-3*, *mcr-4*, *mcr-5*, and *mcr-8* genes as previously described (7, 8). The *mcr-5* gene was detected in one isolate (strain Q4897) which was identified as Cupriavidus gilardii. Cupriavidus gilardii is a glucose-nonfermenting Gram-negative bacterium (GNB) that belongs to the *Burkholderiaceae* family. It was previously classified as Ralstonia gilardii and Wautersia gilardii (9). The gene was fully amplified by standard PCR and sequenced using BigDye terminator chemistry on an ABI 3500xl automated sequencer (Applied Biosystems, Foster City, CA, USA). Sequence analysis confirmed an *mcr-5.1* variant.

The *mcr-5*-positive isolate was examined for its susceptibility to antibiotics using the disc diffusion method, and inhibition zone diameters were interpreted according to the antibiotic committee of the French Society for Microbiology (Société Française de Microbiologie) breakpoints (https://www.sfm-microbiologie.org/wp-content/uploads/2020/04/CASFM2020_Avril2020_V1.1.pdf). In addition, the colistin MIC was determined using the broth microdilution (BMD) method. Our isolate was resistant to ticarcillin, ticarcillin-clavulanate, aztreonam, ertapenem, meropenem, imipenem, gentamicin, fosfomycin, rifampin, and colistin (MIC = 8 μg/ml). The isolate was negative for carbapenemase production using the β-CARBA test (Bio-Rad, Marnes-la-Coquette, France). For whole-genome sequencing (WGS), genomic DNA was extracted using the EZ1 biorobot with the EZ1 DNA tissue kit (Qiagen, Hilden, Germany) and then sequenced on a MiSeq sequencer (Illumina Inc., San Diego, CA, USA) with the Nextera Mate Pair sample preparation kit and Nextera XT Paired End (Illumina). The assembly was performed using a Shovill pipeline (https://github.com/tseemann/shovill). Scaffolds of <800 bp and scaffolds with a depth value lower than 25% of the mean depth were removed. The assembly generated 66 contigs with a total length of 5,335,421 bp and a GC content of 67.3%. The occurrence of antibiotic resistance genes was investigated through the ABRicate function of the Galaxy web platform (https://usegalaxy.org.au/) using ARG-ANNOT, NCBI, CARD, and ResFinder as reference databases with minimum of 70% for identity and coverage. All detected hits are presented in Table 1. In addition to the *mcr-5.1* colistin resistance gene, we identified a class D β-lactamase which was highly similar (90.84% similarity with the reference sequence) to the OXA-837 enzyme and a putative aminoglycoside inactivation enzyme, “*aac(3)-IVa*.” Interestingly, these two antibiotic resistance genes have been found to be well conserved in *C. gilardii* genomes (9). Furthermore, several conserved multidrug efflux pumps were detected, which could explain the multidrug resistance phenotype observed in our isolate.

**TABLE 1:** Antibiotic resistance determinants found in *C. gilardii* Q4897

Gene	% coverage	% identity	Product	Resistance
*mcr-5.1*	100.00	99.94	Phosphoethanolamine-lipid A transferase MCR-5.1	Colistin
*aac(* 3 *)-IVa*	97.81	70.73	Aminoglycoside *N*-acetyltransferase AAC(3)-IVa	Gentamicin
*bla*_OXA-837_	100.00	90.84	Class D β-lactamase OXA-837	β-Lactams
Pseudomonas aeruginosa *emrE*	87.09	72.07	EmrE is a small multidrug transporter that functions as a homodimer and that couples the efflux of small polyaromatic cations from the cell with the import of protons down an electrochemical gradient. Confers resistance to tetraphenylphosphonium, methyl viologen, gentamicin, kanamycin, and neomycin.	Aminoglycoside
*muxB*	96.36	78.44	MuxB is one of the two necessary RND components in the Pseudomonas aeruginosa efflux pump system MuxABC-OpmB.	Aminocoumarin; macrolide; monobactam; tetracycline
*muxC*	72.52	72.05	MuxC is one of the two necessary RND components of the MuxABC-OpmB efflux pumps system in Pseudomonas aeruginosa.	Aminocoumarin; macrolide; monobactam; tetracycline
Pseudomonas aeruginosa *soxR*	89.17	70.24	SoxR is a redox-sensitive transcriptional activator that induces expression of a small regulon that includes the RND efflux pump-encoding operon *mexGHI*-*opmD*. SoxR was shown to be activated by pyocyanin.	Acridine dye; cephalosporin; fluoroquinolone; glycylcycline; penam; phenicol; rifamycin; tetracycline; triclosan
*axyY*	96.02	71.68	AxyY is the periplasmic adaptor protein of the AxyXY-OprZ efflux pump system in *Achromobacter* spp.	Aminoglycoside; cephalosporin; fluoroquinolone; macrolide
*mexC*	82.39	71.92	MexC is the membrane fusion protein of the MexCD-OprJ multidrug efflux complex.	Aminocoumarin; aminoglycoside; cephalosporin; diaminopyrimidine; fluoroquinolone; macrolide; penam; phenicol; tetracycline
*mexD*	97.35	74.52	MexD is the multidrug inner membrane transporter of the MexCD-OprJ complex.	Aminocoumarin; aminoglycoside; cephalosporin; diaminopyrimidine; fluoroquinolone; macrolide; penam; phenicol; tetracycline

**FIG 1:**
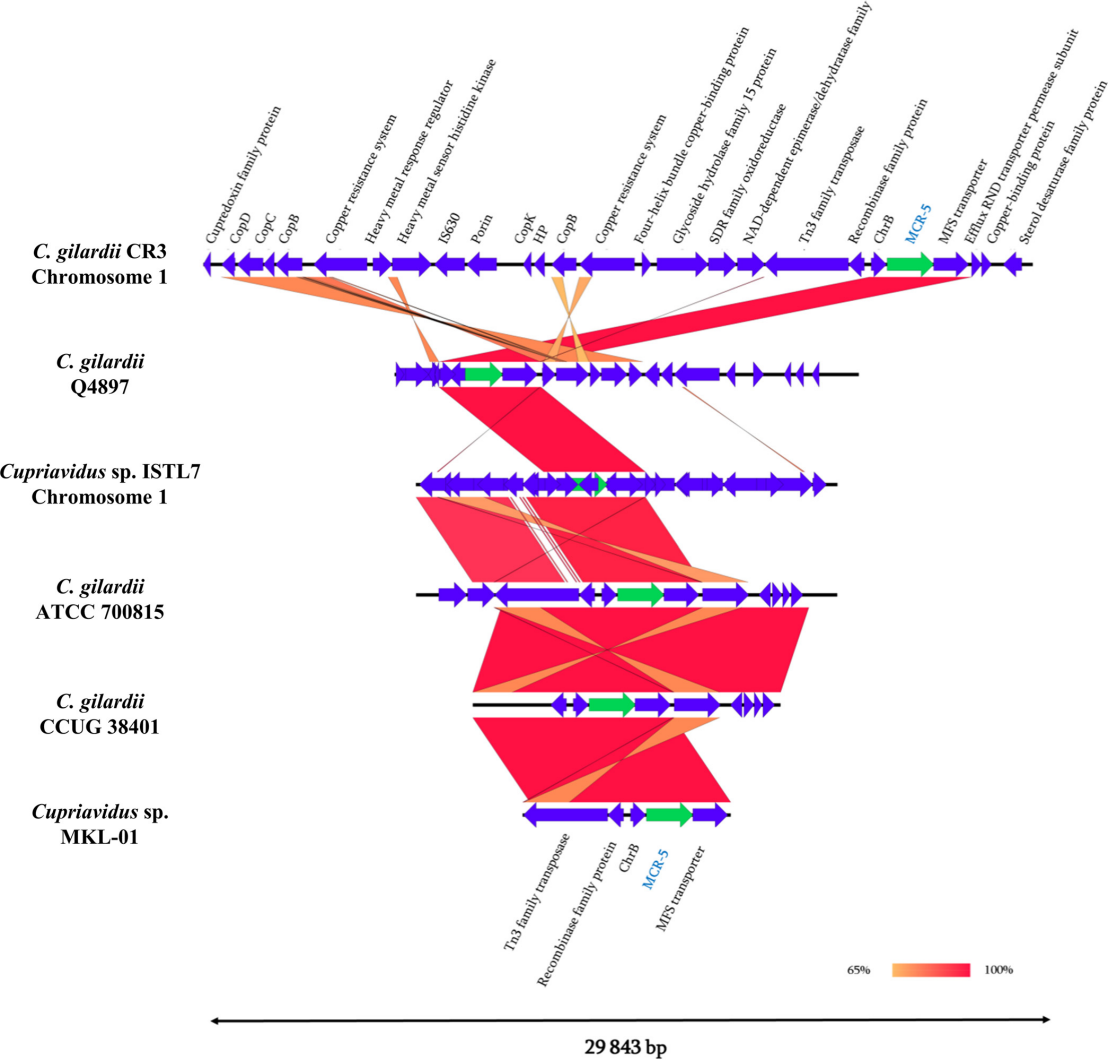
Genomic environment of *mcr-5* genes in *Cupriavidus* genomes. Linear comparison of the *mcr-5*-carrying chromosome fragments of *C. gilardii* strain CR3, *C. gilardii* strain CCUG 38401, *C. gilardii* strain ATCC 700815, *C. gilardii* strain Q4897, *Cupriavidus* sp. strain MKL-01, and *Cupriavidus* sp. strain ISTL7. Boxed arrows represent the position and transcriptional direction of open reading frames. Regions of >99% identity are marked by red shading. MFS, major facilitator superfamily.

In parallel, the *mcr-5* protein reference sequence (WP_053821788.1) from *Proteobacteria* was used to query its presence in all available complete and WGS genomes of *Cupriavidus* from the NCBI database. The *in silico* analysis showed that, of the 127 *Cupriavidus* genomes, five *mcr-5* chromosomic sequences (4% of analyzed genomes) exhibited an identity value at 100% and 100% alignment with the reference sequence. Indeed, it has been suggested that the *mcr-5* gene might have been transferred from environmental *C. gilardii* to Salmonella enterica (10); nevertheless, this gene was identified only in three out of the eight available *C. gilardii* genomes (Table 2) and in two genomes of *Cupriavidus* sp. However, we do not know the susceptibility of these strains to colistin, which could have provided us with more information on the resistance mechanism. In addition, Easyfig v2.2.5 software was used to investigate the genetic environment surrounding the *mcr-5* gene from the five selected genomes as well as from our isolate (Fig. 1).

**TABLE 2:** *mcr-5* detected in *Cupriavidus* genomes (100% of identity and coverage)

No.	Organism	Strain	Genome size (bp)	GC%	Total CDS^*a*^	Assembly level	Isolation source	Geographic location	Accession no.(s)
1	*C. gilardii*	CR3	5,578,743	67.55	4,988	Complete genome	Tar pits	Rancho La Brea, Los Angeles, CA, USA	NZ_CP010516.1; NZ_CP010517.1
2	*C. gilardii*	CCUG 38401	5,792,089	67.4	5,283	Contig	Whirlpool	Missing	NZ_VZOV00000000.1
3	*C. gilardii*	ATCC 700815	5,761,323	67.4	5,253	Contig	Whirlpool	Missing	NZ_JABEMD000000000
4	*C. gilardii*	Q4897	5,335,421	67.3	4,717	Contig	Well water	Batna, Algeria	JAGFTW000000000
5	*Cupriavidus* sp.	MKL-01	5,749,837	67.9	5,043	Scaffold	Blood	Seoul, South Korea	NZ_VWRN00000000
6	*Cupriavidus* sp.	ISTL7	5,578,573	66.75	4,655	Chromosome	Soil	Delhi, India	NZ_CP066227; NZ_CP066228

a CDS, coding DNA sequences.

Our *mcr-5*-positive isolate was recovered from the well supplying the hospital with tap water. Except for drinking, this water is used in all applications requiring water use in the hospital, including cooking, bathing of newborns, cleaning, and hand washing. It is worth mentioning that well water is directly used without any treatment.

The *mcr-5* gene was first described in Salmonella enterica subsp*. enterica* serovar Paratyphi B var. Java *d*Ta+ from Germany, where the authors confirmed that the *mcr-5* gene was located on a 7,337-bp Tn*3*-family transposon harbored by a ColE-type plasmid (10). Interestingly, by using BLASTn search a Tn*3*-family transposon harboring the *mcr-5* gene was also detected in chromosome 1 of a *C. gilardii* strain (CR3) recovered in the United States (10).

*mcr* variants have been previously detected in aquatic environments. *mcr-5* and *mcr-5.4* have been detected by culture-independent methods in a wastewater treatment plant in Germany and in hospital tap water in the Netherlands, respectively (11, 12). In addition, the *mcr-5* gene has been detected in an Enterobacter sp. isolated from hospital sewage in China (13), and an MCR-5.3-producing *Stenotrophomonas* sp. has been isolated from animal waste in China (14). Recently, *mcr-5* has been detected in a *Cupriavidus* sp. closely related to *C. gilardii* isolated from the blood of an immunocompromised patient in South Korea (15).

Members of the *Cupriavidus* genius are known for their resistance to copper and other metals. This might be due to the presence of several metal resistance loci such as *cop* genes, as shown in Fig. 1.

*C. gilardii* is gaining increasing attention as an emerging pathogen, and several studies have reported its role in human infections, including perirectal inflammation, bloodstream infection, muscular abscess, and catheter sepsis (15). In terms of antibiotic resistance, it has been suggested that *C. gilardii* is intrinsically resistant to ertapenem, meropenem, ampicillin, amoxicillin-clavulanate, gentamicin, tobramycin, and streptomycin, while it is susceptible to imipenem and cefotaxime and intermediately resistant to spectinomycin (9). In a study carried out on 39 *Cupriavidus* clinical isolates, including six *C. gilardii* strains, the authors tested the MICs of these strains against 20 antibiotics by BMD, and the results showed that two *C. gilardii* strains were resistant to colistin and four were imipenem resistant. However, the resistance mechanisms were not characterized (16).

Our findings are of great interest, as we present here a potential route for the spread of such resistant organisms in the community, where further investigations and actions are required in order to contain this problem.

### Data availability.

This whole-genome sequence has been deposited at GenBank under accession no. JAGFTW000000000.
